# Special issue: Wearable computing and communication for e-Health

**DOI:** 10.1007/s11517-012-0976-7

**Published:** 2012-10-26

**Authors:** Dario Farina, Ernestina Cianca, Nicola Marchetti, Simone Frattasi

**Affiliations:** 1 Department of Neurorehabilitation Engineering, University Medical Center Göttingen, Göttingen, Germany; 2 Center for TeleInFrastruktur, University of Rome Tor Vergata, Tor Vergata, Italy; 3 CTVR/The Telecommunications Research Centre, Trinity College, Dublin, Ireland; 4 Patrade A/S, Aarhus Municipality, Denmark

Wearable computers and communication devices are computing and communication systems that are body-worn. This technology is used in behavioral modeling, health-monitoring systems, information technologies and media development. Wearable devices are especially useful for applications that require computational and communicational support while the user’s physical and mental capabilities are actively engaged with the physical environment. Wearable technology is a broad topic of research, with areas of study that include user interface design, augmented reality, pattern recognition, targeting specific applications or disabilities. This special issue aimed at collecting contributions on wearable systems with e-health applications. Seven original papers have been selected to cover a wide variety of applications.

The use of smart, wearable technologies can offer ways to reduce the number of nursing staff and costs for care of senior citizens. The paper “User Interaction in Smart Ambient Environment Targeted for Senior Citizen” presents a projection-based display system for elderly people with memory impairments. Thanks to this wearable system, elderly people can interact with the projected user interface by tapping physical surfaces, using them as natural, haptic feedback input interfaces.

Ambient intelligence and wearable computing need innovative hardware and software technologies, including a highly capable, flexible and efficient middleware, allowing for the reuse of existing pervasive applications when developing new ones. In the paper “Towards a Flexible Middleware for Context-aware Pervasive and Wearable Systems”, the authors illustrate how these concepts can be described into a theoretical tool, called Networked Autonomic Machine (NAM), and how they can be implemented into an NAM-based middleware, and evaluated against practical problems of pervasive computing.

A relevant application of wearable computing and communication systems is the long-term monitoring of physiological parameters. In these applications, a key issue is the reduction of the average power consumption of the small wearable device. It is known that a way to reduce the power consumption is by including in the wearable device a low power and real-time compression of the raw physiological data. The paper “Compressive sensing scalp EEG signals: implementations and practical performance” focuses on the use of compressive sensing, which is a new paradigm in the data compression field. In particular, the paper investigates the practical performance of different implementation of compressive sensing systems when applied to scalp EEG signals.

Within the area of physiological data monitoring and its exploitation, another application of wearable e-health is to provide a feedback on physiological parameters to the users. This may be helpful, for example, to reduce the long-term effects of bad posture or body misalignment, which generates pain and potential disabilities. Postural kyphosis in adolescence, which is an excessive rounding of the upper spine, may be one of the effects of poor standing and sitting habits. In the paper “Development of a Smart Garment to Reduce Kyphosis during Daily Living”, a smart garment, consisting of a harness and two data-sensor loggers, was developed to monitor and provide vibration feedback to wearers to improve their posture during daily activities.

Further, the authors of “Measuring Skin Conductance over Clothes” propose a new method that measures skin conductance over clothes to non-intrusively monitor the changes in physiological conditions affecting skin conductance during daily activities. The performance of the proposed method was compared with the traditional Galvanic Skin Response (GSR) measured directly from the fingers. The results proved the effectiveness of the proposed method.

Emotion identification and recognition through wearable devices has become recently a noteworthy research area and it is the topic of the paper “A novel EDA glove based on textile integrated electrodes for affective computing”. The paper describes an experiment aiming at discriminating different affective states using the Electro Dermal Response (EDR). Using non-linear methods, a novel set of features for pattern classification was extracted and used to obtain remarkably successful recognition rates.

Finally, the paper “Kinematic description of soft tissue artifacts: quantifying rigid vs deformation components and their relation with bone motion”, focuses on the use of wearable devices to monitor and analyze human movements. Soft tissue artifacts (STA) are a major source of error in this type of movement analysis. The paper provides the quantitative relation between rigid movements and deformation of marker cluster components of the STA and the results are useful to assess the potential effectiveness of the usual strategies to compensate STA.

The above papers provide a set of representative applications of wearable technology for e-Health. From these contributions it is possible to appreciate the richness in potential applications of such technology but also the many challenges that need to be faced for translating basic research results into practical, daily life applications.

